# Operative Fixation of Lateral Malleolus Fractures With Locking Plates vs Nonlocking Plates: A Systematic Review and Meta-analysis

**DOI:** 10.1177/10711007211040508

**Published:** 2021-09-28

**Authors:** Nesar Ahmad Hasami, Diederik Pieter Johan Smeeing, Albert Frederik Pull ter Gunne, Michael John Richard Edwards, Stijn Diederik Nelen

**Affiliations:** 1 Department of Trauma Surgery, Radboud University Medical Center, Nijmegen, Nijmegen, the Netherlands; 2 Department of Trauma Surgery, Rijnstate Hospital, Arnhem, Gelderland, the Netherlands; 3 Department of Trauma Surgery, University Medical Center Utrecht, Utrecht, the Netherlands

**Keywords:** anatomical locking plate, ankle fracture, fracture fixation, locking plate, nonanatomical locking plate, nonlocking plate, osteoporosis, trauma, osteosynthesis

## Abstract

**Background:**

The exact benefit of locking plates over nonlocking plates in patients with lateral malleolus fractures remains unclear. The primary aim of this study was to compare the functional outcome of locking plates vs nonlocking plates in patients with a lateral malleolus fracture. The secondary aims were to compare the number of complications and hardware removals and to compare whether results differed for older patients and for patients treated with anatomical locking plates.

**Methods:**

The PubMed/MEDLINE, Embase, Cochrane, and CINAHL databases were searched for studies comparing locking plates with nonlocking plates in patients with fixated lateral malleolus fractures. All included studies were assessed on their methodologic quality using the MINORS. Subgroup analyses were performed on older patients and patients treated with anatomical locking plates.

**Results:**

A total of 11 studies were included. The meta-analysis showed that functional outcome did not differ between patients treated with locking plates and nonlocking plates (MD 2.38, 95% CI −2.71 to 7.46). No difference in both complication rate (OR 1.10, 95% CI 0.74-1.63) and the amount of hardware removals (OR 0.77, 95% CI 0.52-1.14) was found. Even after analyzing older patients and patients treated with anatomical locking plates, no benefit was shown.

**Conclusion:**

This meta-analysis demonstrates no clear benefit in selecting locking plates over nonlocking plates in the treatment of lateral malleolus fractures.

**Clinical Relevance:**

Locking plates are increasingly being used in the treatment of lateral malleolus fractures. Biomechanical studies have shown an increased stability with use of locking vs nonlocking plates. This clinical review does not support a benefit of use of locking plates for these fractures.

## Introduction

An ankle fracture is a common type of lower extremity fracture and among the common types of fractures worldwide.^6^ Ankle fractures have a bimodal age distribution with peaks in younger males and older females, and have an incidence rate of 100 to 150 per 100 000 person-year.^13^

In the last decade, the increasing amount of literature on the use of locking plates in ankle fracture surgery suggests an increasing use of this type of plate osteosynthesis in daily practice.^1,2,4,7,8,11,12,17,20,21,23,25,26,28^ Plate osteosynthesis can be carried out using conventional nonlocking plates, such as one-third tubular plates or dynamic compression plates, but plate osteosynthesis can also be carried out with a variety of later developed locking plates, such as locking compression plates or anatomical locking compression plates. Locking plates allow the opportunity to combine dynamic compression principles with internal fixation using locking screws. Because of these additional features, locking plates seem more attractive in terms of biomechanical stability. In patients with osteoporosis or more distal fibular fractures, operative fixation can be more challenging because of limited screw purchase. Therefore, the biomechanical advantages of locking plates seem especially useful in those patients.^18,29^ Moreover, locking plates are considered especially valuable in older patients because they more often have osteoporosis.^14^

Various types of locking plates exist. Locking plates can be divided in anatomical locking plates and nonanatomical locking plates. Anatomical locking plates are precontoured and, therefore, have a better fit on the bone. One might presume that the use of this type of plate could result in a better functional outcome and a lower amount of hardware removals.

Various studies have reported advantages and disadvantages of both types of plating systems. Locking plates have been associated with improved mechanical stability, although these plates are also associated with the need for a longer consolidation period.^3,10,18^ Nonlocking plates are less expensive, but studies suggest that these plates are associated with a higher rate of complications, stability loss, and osteosynthesis-related soft tissue discomfort compared with locking plates.^15,22^ So far, various cohort studies compared locking and nonlocking plates in terms of functional outcome, but a systematic review or meta-analysis on this subject is lacking in recent literature.

The primary aim of this study was to systematically review studies that compared functional outcome in patients with lateral malleolus ankle fractures operatively fixated with locking plates vs fixation with nonlocking plates. The secondary aims were to compare the number of complications, hardware removals, and to compare whether results differed for older patients and patients treated with anatomical locking plates in particular.

## Methods

This systematic review and meta-analysis was conducted following the Preferred Reporting Items for Systematic Reviews and Meta-Analyses (PRISMA) guidelines.^19^

### Search Strategy

On the 2nd of March 2021, the PubMed/Medline, Embase, Cochrane, and CINAHL electronic databases were searched for studies comparing locking plates with nonlocking plates in patients with lateral malleolus fractures requiring operative fixation. Search syntaxes for the different databases were created with assistance of an information specialist at the Radboud University Medical Center and are provided in Appendix 1. No filters or other restrictions were applied in the search. Titles and abstracts of the retrieved records were screened, and duplicates were removed. Potentially suitable studies were read in full by 2 independent reviewers. References of included articles were checked, and citation tracking was performed to identify articles not found in the original literature search.

### Selection Criteria

Both randomized and nonrandomized studies comparing locking plates to nonlocking plates in patients with a lateral malleolus fracture were included. Included patients had to be 16 years or older. Studies had to describe at least 1 of the following outcomes: functional outcome, complication rate, or amount of hardware removals. Exclusion criteria were articles written in a language other than English or Dutch. Animal or cadaver studies, letters, comments, abstracts for conferences, study protocols, and biomechanical studies were also excluded. Differences in article inclusion were discussed, and in case no consensus could be reached a third independent reviewer was consulted to solve disagreements.

### Quality Assessment

Methodologic quality for included studies was independently assessed by 2 reviewers using the Methodological Index for Non-Randomized Studies (MINORS).^27^ The MINORS is a validated instrument designed to assess the methodological quality and clear reporting of observational studies. The MINORS is externally validated using randomized controlled trials and, therefore, also appropriate to assess the quality of randomized controlled trials.^27^ The MINORS assessment criteria are described in Appendix 2.

### Data Extraction

The following data were extracted: first author, year of publication, study design, type of ankle fracture, duration of follow-up, treatment groups, number of patients included, mean age, and male-female ratio. The functional outcome of the ankle, the complication rate, and the amount of hardware removals, including confidence intervals or *P* values, were obtained. Various complications were extracted: wound healing disorders, superficial infections, deep infections, and failure of osteosynthesis. The complications were defined as done by the included study itself.

### Outcome Measures

The primary outcome measure was the functional outcome of the ankle joint after operative treatment with locking plates or nonlocking plates. This was evaluated by including results that used validated functional outcome scores. Both self-reported questionnaires and measure tools for physicians or researchers were defined as appropriate outcomes. The American Orthopaedic Foot & Ankle Society Score (AOFAS score) is an often used rating system that incorporates both subjective and objective information. Patients report their pain, and physicians assess alignment. Scores range from 0 to 100, with healthy ankles receiving 100 points.^16^ Secondary outcome measures included the total complication rate and the number of hardware removals. Finally, subgroup analyses were performed exclusively on older patients (aged 50 years and older) and patients treated with anatomical locking plates compared with nonlocking plates.

### Data Analysis

Statistical analyses and graphical representation were performed using Review Manager software (RevMan v 5.4) provided by the Cochrane Collaboration.^5^ If possible, means or SDs that were not reported in an article were calculated using the available information. Outcomes reported by 2 or more studies were pooled in a meta-analysis. The principal summary measures were odds ratios (ORs) for dichotomous data and mean difference (MD) for continuous data. The assessment of statistical heterogeneity was performed by visual inspection of the forest plots and estimating statistical measures of heterogeneity: Cochran *Q* (chi-square test), *I*^2^, and τ^2^ (tau-square test). The random effects model was used for meta-analyses. A funnel plot was used to detect potential publication bias. The most reported outcome measure in the included studies was put against its standard error.

## Results

### Search

**Figure 1.**
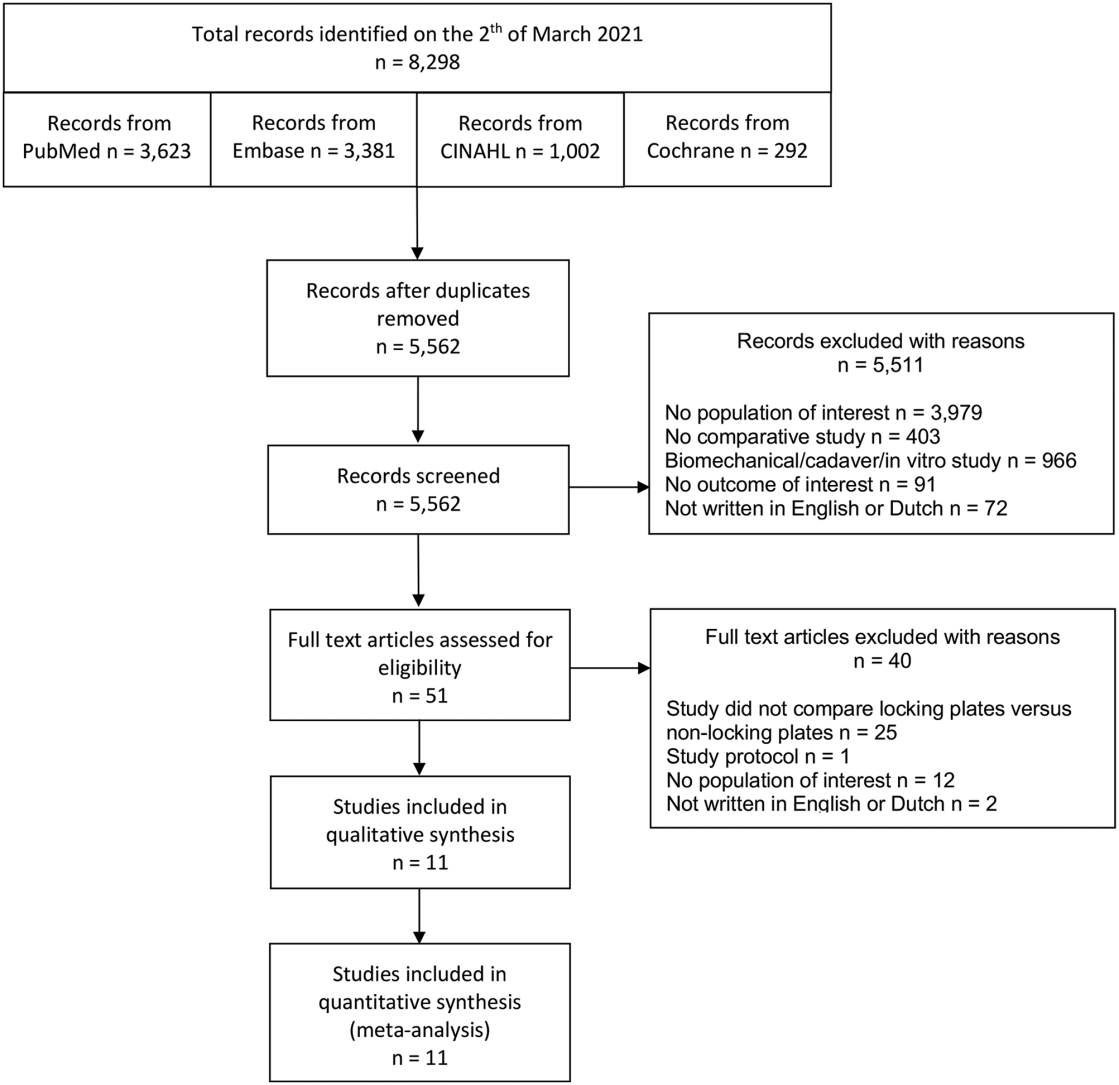
Flowchart of included studies in a systematic review comparing locking plates versus nonlocking plates in operative fixated lateral malleolus fractures.

The electronic search detected a total of 8298 records. After removing duplicates, 5562 records were screened on title and abstract. Most exclusions were studies with no population of interest (n=3979; 72%) (Figure 1). After title and abstract screening, a total of 51 possible relevant studies from the initial search were assessed for eligibility after reading the full text. In the full-text screening, most of the studies excluded were cohort studies describing only 1 group (n=25; 63%). Reference checking and citation tracking did not reveal suitable studies that had not already been found in the initial search. Eleven studies were considered suitable for inclusion in this systematic review and meta-analysis.^4,7,8,11,12,17,20,21,25,26,28^ Ten of the included studies were retrospective cohort studies.^4,7,8,11,12,17,20,21,25,26^ One of the included studies was a randomized controlled trial.^28^

### Quality Assessment

The mean (SD) MINORS score was 16.72 ± 1.42 (range 14-19). Appendix 3 shows the distribution of study quality across the studies. For the retrospective cohort studies, the mean score was 16.50 ± 1.27 (range 14-18). The randomized controlled trial had a score of 19. Visual inspection of the forest plots indicated a moderate statistical heterogeneity.

### Baseline Characteristics

The characteristics of all included studies are described in Table 1. The 10 retrospective cohort studies together included a total of 1321 patients, and the 1 randomized controlled trial included 52 patients. A total of 627 patients were treated with locking plates. The number of patients included per study ranged from 44 to 319. The mean reported follow-up time was 13.0 months (2 studies did not report a follow-up time).

**Table 1.** Baseline Characteristics of Included Studies in a Systematic Review of Locking Plates vs Nonlocking Plates in Operative Fixated Lateral Malleolus Fractures.

First Author	Year	Study Design	Ankle Fracture Types	Follow-up, mo	Treatment Groups	Number of Patients	Age, y, Mean ± SD	Male / Female
Bilgetekin et al^4^	2019	RC	Lateral malleolus fractures	27	Locking anatomical distal fibula plate^a^	25	NR^b^	NR
					One-third tubular plate	37	NR^b^	NR
El Fatayri et al^7^	2019	RC	Lateral malleolus fractures	3	Locking plate^a^	63	49.4 (16.2)	24/39
					Nonlocking plate	42	51.8 (18.7)	18/24
Gentile et al^8^	2015	RC	Lateral malleolus fractures	12	Minifragment fixation with LCP plate or LCP adaption plate	16	51.1 (13.5)	7/9
					Standard fixation with one-third tubular plate	28	41.5 (15.7)	8/20
Herrera-Pérez et al^11^	2017	RC	Lateral malleolus fractures	12	Locking plate	17	73 (5.1)	3/17^c^
					Nonlocking plate	45	72 (5.2)	14/28^c^
Huang et al^12^	2014	RC	Lateral malleolus fractures	12	LCP metaphyseal plate / LCP distal fibula plate^d^	98	48.3 (12.6)	58/40
					One-third tubular plate	49	47.5 (12.7)	29/20
Huang et al^12^	2018	RC	Lateral malleolus fractures	24	Locking plate^d^	58	49 (14.9)	28/30
					Semitubular plate	87	39 (14.7)	46/41
Moriarity et al^20^	2018	RC	Lateral malleolus fractures	3	Locking plate^a^	31	52.6	9/22
					Nonlocking plate	129	39.1	64/65
Moss et al^21^	2017	RC	Lateral malleolus fractures	NR	Contoured locking plate^a^	222	44.4 (13.3)	139/83
					One-third tubular plate	97	37.9 (13.1)	39/58
Schepers et al^25^	2011	RC	Lateral malleolus fractures	NR	Locking plate	40	46.5	19/21
					Nonlocking plate (one-third tubular plate)	165	48.8	85/80
Shih et al^26^	2020	RC	Lateral malleolus fractures	12	Locking plate^a^	34	63.7 (7.9)	10/24
					Nonlocking tubular plate	38	60.2 (7.0)	11/27
Tsukada et al^28^	2013	RCT	Lateral malleolus fractures	12	Locking plate	23	40.7 (14.2)	9/14
					Nonlocking plate	29	41.7 (19.2)	15/14

Abbreviations: NR, not reported; RC, retrospective cohort; RCT, randomized controlled trial.

a Anatomical locking plates were used.

b Median age anatomical distal fibula locking plate group: 40.0 (23-58) years, median age one-third tubular plate group 44.0 (21-59) years.

c Male-female ratio exceeds the total number of enrolled patients.

d Both anatomical and nonanatomical locking plates were used.

### Functional Outcome

**Figure 2.**
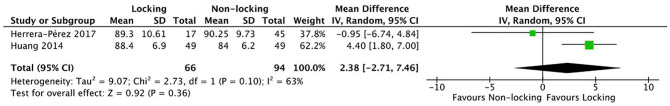
AOFAS (American Orthopaedic Foot & Ankle Society) score in a systematic review comparing locking vs nonlocking plates in operative fixated lateral malleolus fractures.

Of all included studies, 4 (36%) measured the functional outcome of the ankle in patients.^4,11,12,26^ Different functional outcome measure tools were used: Shih et al^26^ used the Foot and Ankle Outcome Score (FAOS), Bilgetekin et al^4^ and Herrera-Pérez et al^11^ used the American Orthopaedic Foot & Ankle Society (AOFAS) score), and Huang et al^12^ used both the Olerud Molander Score (OMS) and the AOFAS score. The follow-up time at which the AOFAS score had been measured was not reported by Bilgetekin et al.^4^ All other studies reported results on the long term (ie, after 6 months), Herrera-Pérez et al^11^ was the only study that reported both short-term (ie, less than 6 months) and long-term scores (Table 2). Huang et al^12^ compared and analyzed functional outcome scores for 3 groups (nonlocking, nonanatomical locking, and anatomical locking). Because mean functional outcome scores for the nonanatomical locking group were not reported by Huang et al,^12^ this group could not be included in the analysis. The reported OMS (locking group: 86.3 ± 6.2, nonlocking group: 82.1 ± 6.9, *P* = .004) and AOFAS scores (locking group: 88.4 ± 6.9, nonlocking group: 84.0 ± 6.2, *P* = .002) by Huang et al^12^ were significant in favor of locking plates. Bilgetekin et al^4^ reported a median AOFAS score (locking group: 85.0; nonlocking group: 87.0, *P =* .339) and not a mean score and therefore could not be included in the analysis. Shih et al^26^ reported a significant difference in FAOS in favor of locking plates (431.1 ± 31.2 vs 403.7 ± 38.1, *P =* .002). Owing to the limited availability of studies, only the analysis on long-term AOFAS score was possible. No difference was found (MD 2.38, 95% CI −2.71 to 7.46) (Figure 2).

**Table 2.** Study Outcomes and Results in a Systematic Review Comparing Locking Plates vs Nonlocking Plates in Operative Fixated Lateral Malleolus Fractures.

Study	Functional Scores Short Term (<6 mo)		Functional Scores Long Term (>6 mo)		Complications, n (%)										Amount of Hardware Removals, n (%)	
	Locking	Nonlocking	Locking	Nonlocking	Wound Healing Disorders		Superficial Infections		Deep Infections		Failure of Osteosynthesis		Total		Locking	Nonlocking
					Locking	Nonlocking	Locking	Nonlocking	Locking	Nonlocking	Locking	Nonlocking	Locking	Nonlocking		
Bilgetekin et al^4^	NR	NR	AOFAS: 85.0 (median)	AOFAS: 87.0 (median)	NR	NR	1 (4.0)	0	0	0	0	0	1 (4.0)	0	0	2 (5.4)
El Fatayri et al^7^	NR	NR	NR	NR	NR	NR	5 (7.94)	1 (2.38)	2 (3.17)	4 (9.52)	NR	NR	7 (11.11)	5 (11.90)	25 (39.68)	13 (30.95)
Gentile et al^8^	NR	NR	NR	NR	NR	NR	1 (6.25)	2 (7.14)	1 (6.25)	0	NR	NR	4 (25.00)	2 (7.14)	2 (12.5)	5 (17.9)
Herrera-Pérez et al^11^	AOFAS: 85.73 ± 11.33	AOFAS: 88.41 ± 11.33	AOFAS: 89.30 ± 10.61	AOFAS: 90.25 ± 9.73	4 (8.89)	1 (5.88)	2 (11.76)	1 (2.22)	NR	NR	1 (5.88)	0	4 (23.53)	7 (15.55)	NR	NR
Huang et al^12^	NR	NR	OMS: 86.3 ± 6.2* AOFAS: 88.4 ± 6.9*	OMS: 82.1 ± 6.9 AOFAS: 84.0 ± 6.2	NR	NR	0	1 (2.04)	0	0	0	0	1 (1.02)	1 (2.04)	NR	NR
Lyle et al^17^	NR	NR	NR	NR	1 (1.7)	6 (6.9)	5 (8.6)	4 (4.6)	2 (3.4)	2 (2.3)	2 (3.4)	4 (4.6)	10 (17.24)	16 (18.39)	7 (12.07)	19 (21.83)
Moriarity et al^20^	NR	NR	NR	NR	NR	NR	1 (3.23)	5 (3.97)	0	0	NR	NR	5 (19.23)	22 (17.46)	2 (7.69)	9 (7.14)
Moss et al^21^	NR	NR	NR	NR	NR	NR	NR	NR	NR	NR	1 (1.03)	4 (1.80)	7 (7.22)	7 (3.15)	9 (9.3)	5 (2.3)
Schepers et al^25^	NR	NR	NR	NR	NR	NR	NR	NR	NR	NR	NR	NR	7 (17.5)	9 (5.5)	11 (27.5)	45 (27.3)
Shih et al^26^	NR	NR	FAOS: 431.1 ± 31.2	FAOS: 403.7 ± 38.1	2 (5.88)	2 (5.26)	1 (2.94)	1 (2.63)	0	0	0	0	3 (8.82)	3 (7.89)	6 (17.65)	16 (42.11)
Tsukada et al^28^	NR	NR	NR	NR	0	1 (3.45)	0	2 (6.89)	0	0	0	0	0	3 (10.34)	NR	NR

Abbreviations: AOFAS, American Orthopaedic Foot & Ankle Society; FAOS, Foot and Ankle Outcome Score; NR, not reported; OMS, Olerud Molander Score.

* Functional outcome scores of Huang et al^12^ (both OMS and AOFAS) in the locking group are means of 49 patients.

### Complications and Hardware Removal

All of the included studies reported complications. All complications were registered during the reported follow-up time of each study. In general, the definition of included complications was comparable among studies.

**Figure 3.**
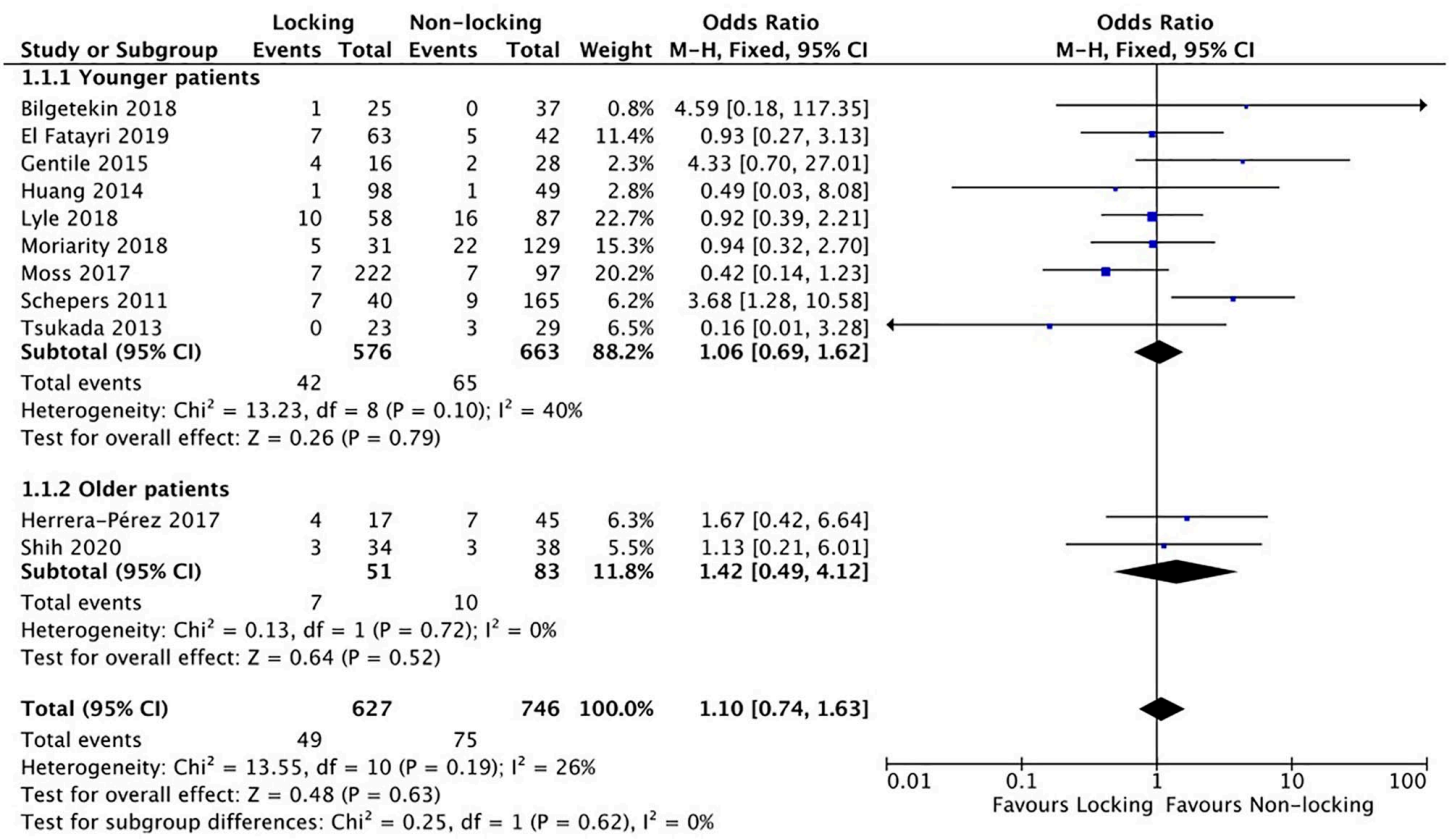
Complications in a systematic review of comparing locking vs nonlocking plates in operative fixated lateral malleolus fractures.

When patients with a wound infection were treated with oral antibiotics, it was defined as a superficial infection. When antibiotics were given intravenously, the infection was defined as a deep infection. The total complication rate for each study was the sum of all reported complications including wound healing disorders, superficial infections, deep infections, and failure of osteosynthesis (Table 2). Two studies (Herrera-Perez et al^11^; Gentile et al^8^) showed an inconsistency between the reported amount of infections in the tables and text.^11,26^ For analysis the highest reported infection rate was used. Analysis showed no difference in the total number of complications (OR 1.10, 95% CI 0.74-1.63) (Figure 3).

**Figure 4.**
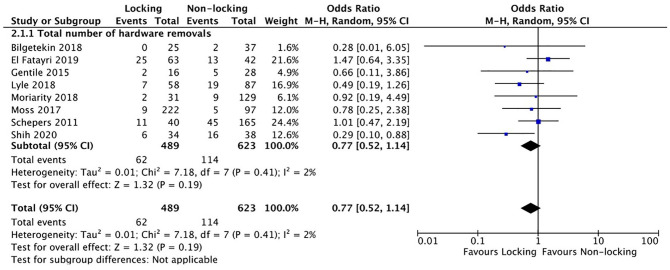
Hardware removals in a systematic review of comparing locking vs nonlocking plates in operative fixated lateral malleolus fractures.

Of all included studies 8 studies (73%) reported the percentage of hardware removals (Table 2). Hardware removal was not compulsory in any of the included studies and only performed if symptomatic. No difference was found in the amount of hardware removals (OR 0.77, 95% CI 0.52-1.14) (Figure 4).

### Older Patients

Two studies (Herrera-Pérez et al^11^; Shih et al^26^) mainly focused on older patients. Together they included a total of 134 patients, of which 51 patients were treated with locking plates.^11,26^ Both studies had a follow-up time of 12 months. Herrera-Pérez et al^11^ only included patients of 65 years and older. The mean (±SD) age of patients treated with locking plates in the study of Herrera-Pérez et al^11^ was 73 ± 5.1 years, and for patients treated with nonlocking plates, this was 72 ± 5.4 years. Shih et al^26^ included patients of 50 years and older (mean age locking group: 63.7 ± 7.9 years; mean age nonlocking group 60.2 ± 7.0 years). Herrera-Pérez et al^11^ measured functional outcome with the AOFAS score and found no difference after 6 months (locking group: 85.73 ± 11.33; nonlocking group: 88.41 ± 11.33, *P* = .37) and 12 months (locking group: 89.30 ± 10.61; 90.25 ± 9.73, *P* = .41). Shih et al^26^ measured functional outcome with the FAOS score and was the only article that focused on older patients that reported a difference (locking group: 431.1 ± 31.2; nonlocking group: 403.7 ± 38.1, *P* < .002). Both articles reported the amount of complications. No difference was found for the amount of complications in older patients (OR 1.42, 95% CI 0.49-4.12) (Figure 3). Shih et al^26^ was the only article that focused on older patients that reported the amount of hardware removals. They reported a statistically significant difference (*P =* .039) with 6 hardware removals in the locking group (17.65%) and 16 removals in the nonlocking group (42.11%).

### Anatomical Locking Plates

Of all the included studies, 7 used anatomical locking plates and 4 used nonanatomical locking plates. Huang et al^12^ and Lyle et al^17^ used both anatomical and nonanatomical plates but reported outcomes for both types of plates separately and therefore could be included in the subgroup analysis. In the subgroup analysis, a total of 460 patients were treated with anatomical locking plates. No subgroup analysis on functional outcome could be performed because of limited studies reporting on the same functional outcome measure. Analysis of the 7 studies on the amount of complications between anatomical locking plates and nonlocking plates showed no difference (OR 0.85, 95% CI 0.52-1.40) (Appendix 4). Analysis of 7 studies on amount of hardware removals between anatomical locking plates and nonlocking plates showed no difference (OR 0.58, 95% CI 0.27-1.27) (Appendix 5).

### Assessment of Publication Bias

**Figure 5.**
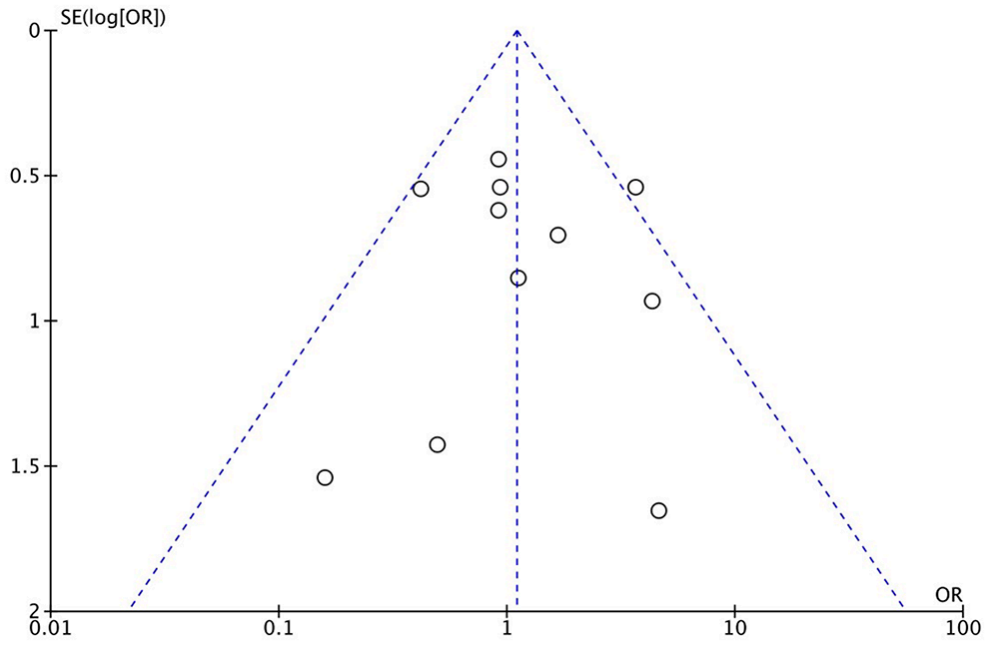
Funnel plot of studies including complications in a systematic review comparing locking vs nonlocking plates in operative fixated lateral malleolus fractures.

The primary outcome (functional outcome) was only reported by 3 articles in the same outcome measure; therefore, publication bias was assessed on the secondary outcome measure. The complication rate was reported by all included articles. A funnel plot with the odds ratios of the studies is shown in Figure 5. The funnel plot asymmetry analysis showed relative symmetry, indicating no evidence or a very low risk of existing publication bias in this systematic review and meta-analysis.

## Discussion

The results of this systematic review and meta-analysis of 11 both randomized and non-randomized studies showed that the use of locking plates in operatively fixated lateral malleolus fractures does not give a better outcome in terms of ankle function, postoperative complication rate, or the amount of hardware removals.

Biomechanical studies show that, compared with nonlocking plates, locking plates give more mechanical stability in surgical fracture treatment.^18,29^ One might therefore expect that the use of these locking plates would also result in fewer complications such as failure of osteosynthesis or nonunion. Theoretically, the increased mechanical stability should lead to a better functional outcome of the ankle. This systematic review not only showed that no difference existed in the amount of complications but also showed no difference in functional outcome. Owing to limited events, it was impossible to perform a reliable meta-analysis on separate complications such as failure of osteosynthesis. Schepers et al^25^ was the only study to report a statistically significant difference in complication rate due to more wound complications occurring in the locking group, compared with the nonlocking group (17.5% vs 5.5%; *P* = .019). Based on the diamond concept, a conceptual framework for a successful bone repair response, mechanical stability is one of the essential factors in bone healing.^9^ Potentially, increased stability due to locking plates, might result in fewer bone healing disorders. Nonunions or delayed unions were not reported by all included studies and therefore could not be included in the analysis of total complication rates per study. The studies that reported the amount of nonunions or delayed unions did not find significant differences between locking and nonlocking plates.^2,8,11,12,20^ The study by Tsukada et al^28^ was a randomized controlled trial with union rate as the primary outcome and did not find statistically significant differences after 3 months (*P* = .22), 6 months (*P =* .18), and 12 months (*P =* .47) between locking plates and nonlocking plates.^28^ The hypothesis that a locking plate improved functional outcome was not supported by this review. Of the 4 articles reporting functional outcome of the ankle, the studies of Huang et al^12^ and Shih et al^26^ showed a better functional outcome for patients treated with locking plates. Bilgetekin et al^4^ and Herrera-Pérez et al^11^ found no differences in functional outcome. No convincing arguments why the studies differed in results were found. Because of the inclusion criteria, the studies of Bilgetekin et al^4^ and Shih et al^26^ were not included in the meta-analysis. This could have tilted the results; however, the chance and clinical relevance seem small as Bilgetekin et al^4^ report no significant difference in functional outcome and the reported difference of Shih et al^26^ is only 27.4 points on a scale of 500.

Locking plates have previously been associated with improved biomechanical stability, especially in osteoporotic bone.^18^ This is why, nowadays, orthopaedic trauma surgeons regularly use locking plates in older patients to make the most stable osteosynthesis. Nevertheless, the subgroup analysis of this systematic review showed no benefit of locking plates in terms of postoperative complications, such as failure of osteosynthesis.

Among other reasons, surgeons use anatomical plates because of the assumption that a better fit on the fibula prevents further surgery for hardware removal. This review, and the subgroup analysis in particular, showed that the use of locking plates does not result in fewer hardware removals. The removal rate therefore seems more related to the thickness of the plate than to the locking principle. Shih et al^26^ was the only study to report significantly fewer hardware removals when locking plates were used. No convincing arguments were found why the results of this study differed from the results of the other studies that were included in this systematic review.

Strengths of this systematic review and meta-analysis are that due to the broad search string in different databases and the inclusion of all relevant study designs, a large number of articles were found and could be included. The overall quality of included studies is good, and the funnel plot suggests a low risk of publication bias. A limitation of this study is that most included studies are retrospective studies; possibly, the treating surgeon made a decision to use a specific plate based on bone quality and fracture configuration. This expert opinion could be one of the reasons why these plates perform equally in terms of functional outcome, complication rate, and hardware removal. Second, all studies are lacking information on bone density. The studies on older patients used age as a proxy for osteoporosis, but the exact role of locking plates in osteoporosis remains unclear. Moreover, limited evidence on treatment of ankle fractures in elderly patients is available. This study only included 2 studies that focused on older patients, and both studies used different outcome measures for the functional outcome. Other limitations are the fact that the AOFAS score is a nonvalidated functional outcome scoring system, and the fact that analysis of the various complications separately was impossible owing to limited events.

As there is little evidence so far, future research on locking plates should focus especially on elderly patients and patients with osteoporosis. To our knowledge no studies have been performed on a geriatric population. Nor are studies done in patients with well-studied and defined osteoporosis. Ankle fracture treatment in geriatric patients (with osteoporosis) will be of growing importance, considering the aging population.^24^ Furthermore the role of locking plates in the treatment of more distal (multifragmentary) and nonunion ankle fractures remains unclear. Patients could potentially benefit from locking plates for these indications. Therefore, the use of locking plates needs to be more thoroughly researched specifically for patients with these indications. Nevertheless, the results of this systematic review and meta-analysis do not demonstrate any advantages of the use of locking plates in ankle fractures in a general population. Given the results of this study, the increased costs that come with use of locking plates cannot be supported. Moreover, it seems that there is no reason to choose locking plates over nonlocking plates in the treatment of lateral malleolus fractures.

## Conclusion

This systematic review and meta-analysis showed that the theoretical benefit of locking plates in the operative treatment of lateral malleolus fractures is not supported by clinical evidence. Given the available evidence on locking plates, no clinically relevant benefit of locking plates in the operative treatment of lateral malleolus fractures was found. Even in the treatment of older patients or by using anatomical locking plates, this review found no clear benefit of locking plates in terms of ankle function, complication rate, and amount of hardware removals.

## Supplemental Material

sj-docx-1-fai-10.1177_10711007211040508 – Supplemental material for Operative Fixation of Lateral Malleolus Fractures With Locking Plates vs Nonlocking Plates: A Systematic Review and Meta-analysis Click here for additional data file. Supplemental material, sj-docx-1-fai-10.1177_10711007211040508 for Operative Fixation of Lateral Malleolus Fractures With Locking Plates vs Nonlocking Plates: A Systematic Review and Meta-analysis by Nesar Ahmad Hasami, Diederik Pieter Johan Smeeing, Albert Frederik Pull ter Gunne, Michael John Richard Edwards and Stijn Diederik Nelen in Foot & Ankle International

sj-pdf-1-fai-10.1177_10711007211040508 – Supplemental material for Operative Fixation of Lateral Malleolus Fractures With Locking Plates vs Nonlocking Plates: A Systematic Review and Meta-analysis Click here for additional data file. Supplemental material, sj-pdf-1-fai-10.1177_10711007211040508 for Operative Fixation of Lateral Malleolus Fractures With Locking Plates vs Nonlocking Plates: A Systematic Review and Meta-analysis by Nesar Ahmad Hasami, Diederik Pieter Johan Smeeing, Albert Frederik Pull ter Gunne, Michael John Richard Edwards and Stijn Diederik Nelen in Foot & Ankle International
