# Supplementary material for: Operative Fixation of Lateral Malleolus Fractures With Locking Plates vs Nonlocking Plates: A Systematic Review and Meta-analysis
Source: Foot Ankle Int. 2021 Sep 28;43(2):280–90. doi: 10.1177/10711007211040508 (PMC8841627; doi:10.1177/10711007211040508)
Supplement: sj-docx-1-fai-10.1177_10711007211040508 – Supplemental material for Operative Fixation of Lateral Malleolus Fractures With Locking Plates vs Nonlocking Plates: A Systematic Review and Meta-analysis [file sj-docx-1-fai-10.1177_10711007211040508.docx]

**Appendix 1 Search strings**

The following databases were all searched on the 2^th^ of March 2021 using the following search syntaxes:

Pubmed/Medline (n = 3723)

("Ankle Fractures"[MeSH Terms] OR "Fibula/injuries"[Mesh] OR "Tibia/injuries"[Mesh] OR "Fibula/surgery"[Mesh] OR "Tibia/surgery"[Mesh] OR "Ankle Injuries"[Mesh] OR Ankle*[tiab] OR fibula [tiab] OR tibia [tiab] OR malleol* [tiab] OR Trimalleolar[tiab] OR Bimalleolar[tiab]) AND ("Bone Plates"[Mesh] OR (Locking[tiab] OR locked[tiab] OR nonlocking[tiab] OR nonlocked[tiab] OR Non-locking[tiab] OR Non-locked[tiab] OR contour*[tiab] OR compress*[tiab] OR conventional[tiab]) AND (plate*[tiab] OR plating[tiab] OR fixat*[tiab] OR osteosynthes*[tiab]))

Embase (n = 3037)

(ankle fracture/ or exp malleolus fracture/ or exp fibula fracture/ or exp fibula/su or tibia fracture/ or exp distal tibia fracture/ or Tibia/su or exp distal tibia/ or (Ankle* or fibula or tibia or malleol* or Trimalleolar or Bimalleolar).ti,ab,kw.) and (exp bone plate/ or ((Locking or locked or nonlocking or nonlocked or Non-locking or Non-locked or contour* or compress* or conventional) adj3 (plate* or plating or fixat* or osteosynthes*)).ti,ab,kw,dq,my.)

Cochrane (n = 262)

((Ankle* OR fibula OR tibia OR malleol* OR Trimalleolar OR Bimalleolar):ti,ab,kw) AND (((Locking OR locked OR nonlocking OR nonlocked OR Non-locking OR Non-locked OR contour* OR compress* OR conventional) AND (plate* OR plating OR fixat* OR osteosynthes*)):ti,ab,kw)

*Word variations have been searched*

CINAHL (n = 1024)

(TI ( ((Locking OR locked OR nonlocking OR nonlocked OR Non-locking OR Non-locked OR contour* OR compress* OR conventional) AND (plate* OR plating OR fixat* OR osteosynthes*)) ) OR AB ( ((Locking OR locked OR nonlocking OR nonlocked OR Non-locking OR Non-locked OR contour* OR compress* OR conventional) AND (plate* OR plating OR fixat* OR osteosynthes*)) )) AND ((MH "Ankle Fractures") OR (MH "Fibula Fractures") OR (MH "Tibial Fractures") OR (MH "Fibula/SU/IN") OR (MH "Tibia/SU/IN") OR TI ( Ankle* OR fibula OR tibia OR malleol* OR Trimalleolar OR Bimalleolar ) OR AB ( Ankle* OR fibula OR tibia OR malleol* OR Trimalleolar OR Bimalleolar ))

**Appendix 2 MINORS assessment criteria**

| **Methodological items** | **2** | **1** | **0** |
| --- | --- | --- | --- |
| **A clearly stated aim** | Aim or hypothesis including outcomes have been reported | Aim or hypothesis have been reported without a clear outcome | Not reported |
| **Inclusion of consecutive patients** | Explicit inclusion and exclusion criteria have been reported | Unclear or poor description inclusion and exclusion criteria have been reported | Not reported |
| **Prospective collection of data** | Prospective | Retrospective | Not reported |
| **Endpoints appropriated to the aim of the study** | Outcomes are appropriate to the aim of the study | Outcomes are not appropriate to the aim of the study | Not reported |
| **Unbiased assessment of the study endpoint** | Blind evaluation of objective outcomes and double-blind evaluation of subjective outcomes | One or more outcomes have been blinded | Blinding has not been performed or is not reported |
| **Follow-up period appropriate to the aim of the study** | ≥ 1 year | < 1 year | Not reported |
| **Loss to follow up** | ≤ 5% | > 5% and ≤ 20% | Not reported or more than 20% |
| **Prospective calculation of the study size** | Power analysis has been performed | Explanation for the number of included patients without a power analysis | Not reported or not performed |
| **An adequate control group** | Locking plates compared with non-locking plates | Not applicable | Not reported |
| **Contemporary group** | Study group and controls have been managed during the same time period | Study groups and controls have not been managed during the same time period | Not reported or unclear description |
| **Baseline equivalence of groups** | Baseline characteristics have been described for both groups and are comparable | Baseline characteristics have not been described thoroughly or are not comparable | Not reported |
| **Adequate statistical analyses** | Statistical analysis has been described including type of test | Inadequate statistical analysis | Not reported |

**Appendix 3 Quality assessment of all included studies in a systematic review of comparing locking plates versus non-locking plates in operative fixated lateral malleolus fractures**

| **Criteria** | **Bilgetekin et al.** | **Fatyri et al.** | **Gentile et al.** | **Herrera-Pérez et al.** | **Huang et al.** | **Lyle et al.** | **Moriarity et al.** | **Moss et al.** | **Schepers et al.** | **Shih et al.** | **Tsukada et al.** |
| --- | --- | --- | --- | --- | --- | --- | --- | --- | --- | --- | --- |
| A clearly stated aim | 2 | 2 | 2 | 2 | 1 | 1 | 2 | 2 | 2 | 2 | 1 |
| Inclusion of consecutive patients | 2 | 2 | 2 | 2 | 2 | 2 | 2 | 2 | 2 | 2 | 1 |
| Prospective collection of data | 1 | 1 | 1 | 1 | 1 | 1 | 1 | 1 | 1 | 1 | 2 |
| Endpoints appropriate to the aim of the study | 2 | 2 | 2 | 2 | 2 | 1 | 2 | 2 | 2 | 2 | 2 |
| Unbiased assessment of the study endpoint | 0 | 0 | 0 | 0 | 0 | 0 | 0 | 0 | 0 | 0 | 2 |
| Follow-up period appropriate to the aim of the study | 2 | 1 | 2 | 2 | 2 | 2 | 1 | 0 | 0 | 2 | 2 |
| Loss to follow-up ≤ 5% | 0 | 1 | 0 | 0 | 0 | 0 | 2 | 2 | 0 | 0 | 1 |
| Prospective calculation of the study size | 1 | 1 | 1 | 0 | 0 | 0 | 0 | 1 | 1 | 0 | 0 |
| An adequate control group | 2 | 2 | 2 | 2 | 2 | 2 | 2 | 2 | 2 | 2 | 2 |
| Contemporary groups | 2 | 2 | 2 | 2 | 2 | 2 | 2 | 2 | 2 | 2 | 2 |
| Baseline equivalence of groups | 2 | 2 | 1 | 0 | 2 | 1 | 2 | 1 | 2 | 2 | 2 |
| Adequate statistical analysis | 2 | 2 | 2 | 2 | 2 | 2 | 2 | 2 | 2 | 2 | 2 |
| **Total quality score MINORS** | **17** | **18** | **17** | **15** | **16** | **14** | **18** | **17** | **16** | **17** | **19** |

**Appendix 4** **Complications in a systematic review of comparing locking versus non-locking plates in operative fixated lateral malleolus fractures with anatomical locking plates**

**Appendix 5** **Hardware removals in a systematic review of comparing locking versus non-locking plates in operative fixated lateral malleolus fractures with anatomical locking plates**
